# Comparison of outcomes of tyrosine kinase inhibitor in first- or second-line therapy for advanced non-small-cell lung cancer patients with sensitive EGFR mutations

**DOI:** 10.18632/oncotarget.12035

**Published:** 2016-09-15

**Authors:** Jianlin Xu, Xueyan Zhang, Haitang Yang, Guozheng Ding, Bo Jin, Yuqing Lou, Yanwei Zhang, Huimin Wang, Baohui Han

**Affiliations:** ^1^ Department of Pulmonary, Shanghai Chest Hospital, Shanghai Jiaotong University, Shanghai, China; ^2^ Department of Thoracic Surgery, Shanghai Chest Hospital, Shanghai Jiaotong University, Shanghai, China; ^3^ Department of Pulmonary, Anqing Municipal Hospital, Anhui, China

**Keywords:** EGFR TKI, NSCLC, first-line, second-line

## Abstract

Direct comparisons between the use of first- and second-line EGFR tyrosine kinase inhibitor (TKI) in patients with sensitive EGFR mutations are limited. A total of 264 advanced non-small-cell lung cancer (NSCLC) patients with sensitive mutations received EGFR TKI therapy as the first-line therapy, and a total of 187 patients received TKI as the second-line therapy at Shanghai Chest Hospital. First-line EGFR TKI therapy [12.9 months, 95% confidence interval (CI), 10.7–15.2] provided longer progression-free survival (PFS) than did second-line EGFR TKI therapy (9.0 months, 95% CI, 7.7–10.2) [hazard ratio (HR): 0.78, *P* = 0.034]. The objective response rate (ORR) of first-, and second-line TKI therapy were 67.8% (159/233) and 55.6% (94/169), respectively (*P* = 0.001). The overall survival (OS) for patients (*n* = 141) receiving first-line TKI followed by second-line chemotherapy were longer than those for patients (*n* = 187) receiving first-line chemotherapy followed by second-line TKI (HR: 0.69, *P* = 0.02).

Compared with second-line TKI, first-line therapy achieved a significant and longer PFS, and higher ORR in the sensitive EGFR mutated NSCLC patients. The therapeutic strategy of using TKI followed by chemotherapy achieved longer OS than that using chemotherapy followed by TKI.

## INTRODUCTION

Worldwide, lung cancer is the most frequently diagnosed cancer. Non-small-cell lung cancer (NSCLC) constitutes approximately 85%–90% of all lung cancers [1, 2]. Platinum-based chemotherapy provides a survival benefit for patients with advanced lung cancer; however, most patients cannot survive more than 1 year [3].

The recognition of a subgroup of patients with NSCLC harboring mutations of EGFR that exhibit a favorable response to tyrosine kinase inhibitor (TKI) has changed the treatment patterns and outcomes of NSCLC [4–6]. Several randomized studies demonstrated that, for EGFR mutated NSCLC, first-line TKI therapy could provide higher tumor response rates (RR) and longer progression-free survival (PFS) than chemotherapy. However, most of these studies failed to demonstrate improvement in overall survival (OS) [7–12]. The failure might be explained by the high proportion of patients from the chemotherapy arm crossing over to the EGFR TKI arm on progression [13, 14]. Patients with EGFR mutations may also benefit from second- or third-line EGFR TKI therapy. Therefore, this raises the question of whether TKI is more effective in EGFR mutated NSCLC patients as a first-line therapy or is equally effective when administered as a second-line therapy [15].

In this study, we summarized the clinical data from patients at Shanghai Chest Hospital in order to compare the outcomes from TKIs used in first- or second-line therapy for advanced NSCLC patients with sensitive EGFR mutations.

## RESULTS

A total of 457 patients with sensitive EGFR mutations (19del or 21L858R) who received TKI therapy were identified (264 patients received TKI and 193 received chemotherapy as the first-line therapy). Among patients treated with TKI as the first-line treatment, 141 received chemotherapy as the second-line therapy. Among patients treated with chemotherapy as the first-line treatment, 187 received TKI as the second-line therapy. Demographic data of patients receiving TKI as the first- or second-line therapy are shown in Table 1.

**Table 1:** Demographic data of patients receiving TKI as first-, or second-line therapy

Characteristic	First-line TKI (*n* = 264)	Second-line TKI (*n* = 187)	*P*
Median age (range)	63 (32–86)	61 (30–81)	
≥ 60	164 (62.1%)	103 (55.1%)	0.134
< 60	100 (37.9%)	84 (44.9%)	
Gender			
Male	103 (39.0%)	84 (44.9%)	0.210
Female	161 (61.0%)	103 (55.1%)	
Smoking status			
Smoker	58 (22.0%)	45 (24.1%)	0.602
Never-smoker	206 (78.0%)	142 (75.9%)	
Histology			
Adeno	249 (94.3%)	161 (86.1 %)	0.003
Others	15 (5.7%)	26 (13.9%)	
Types of EGFR TKI			
Erlotinib	68 (25.8%)	38 (20.3%)	0.031
Gefitinib	147 (55.7%)	95 (50.8%)	
Icotinib	49 (18.6%)	54 (28.9%)	
Mutation type			
19 del	145 (54.9%)	94 (50.3%)	0.329
21 L858R	119 (45.1%)	93 (49.7%)	
PS			
0–1	242 (91.7%)	183 (97.9%)	0.021
≥ 2	18 (6.8%)	4 (2.1%)	

Abbreviation: TKI, tyrosine kinase inhibitor.

### Efficacy of treatments

**Figure 1:**
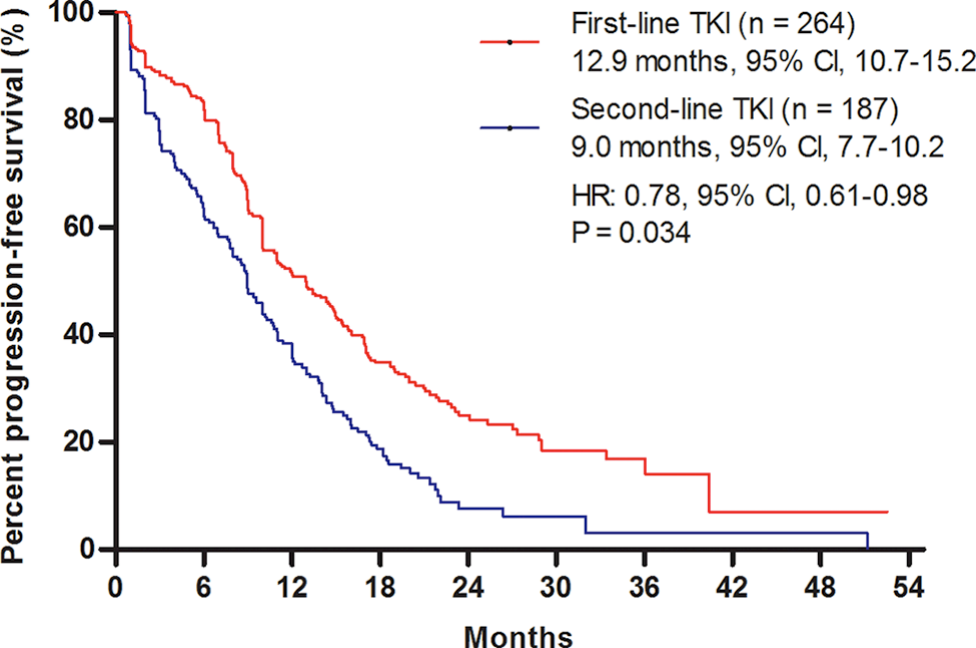
Comparison of progression-free survival (PFS) Kaplan–Meier survival curves for PFS analyses between first- and second-line TKI therapies. TKI, tyrosine kinase inhibitor.

First-line EGFR TKI therapy (12.9 months, 95% CI, 10.7–15.2) provided a longer PFS than did second-line EGFR TKI therapy (9.0 months, 95% CI, 7.7–10.2; HR, 0.78, 95% CI, 0.61–0.98; *P* = 0.034) (Figure 1). There were 402 patients available for an analysis of best response rate from TKI therapy. The overall response rates (ORRs) of first- and second-line TKI therapy for sensitive EGFR mutated patients were 67.8% (159/233), and 55.6% (94/169), respectively (*P* = 0.001) (Table 2).

**Table 2:** Best response to EGFR TKI therapy

Response rate (RR)	First-line TKI therapy (*n* = 233)	Second-line TKI therapy (*n* = 169)
Complete response (CR), *n* (%)	11 (4.7%)	2 (1.2%)
Partial response (PR), *n* (%)	147 (63.1%)	92 (54.4%)
Stable disease (SD), *n* (%)	58 (24.9%)	55 (30.8%)
Progressive disease (PD), *n* (%)	17 (7.3%)	23 (13.6%)
Objective response rate (ORR), *n*(%)	158 (67.8%)	94 (55.6%)

Abbreviation: TKI, tyrosine kinase inhibitor.

**Figure 2:**
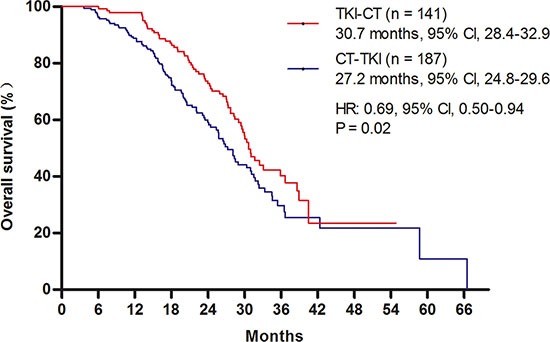
Comparison of overall survival (OS) Kaplan–Meier survival curves for OS analyses between first- and second-line TKI therapies. TKI, tyrosine kinase inhibitor.

The OS analyses were conducted comparing two defined groups of patients: patients treated in sequence with first-line chemotherapy followed by TKI in the second-line (CT-TKI) (*n* = 187), and patients treated with first-line TKI followed by chemotherapy in the second-line (TKI-CT) (*n* = 141). A comparison of baseline characteristic of CT-TKI and TKI-CT cohorts are shown in Supplementary Table S1 and Supplementary Table S2. Our results demonstrated that the OS for the TKI-CT group (30.7 months, 95% CI, 28.4–32.9) was longer than those in the CT-TKI group (27.2 months, 95% CI, 24.8–29.6) (HR, 0.69, 95% CI, 0.50–0.94; *P* = 0.02) (Figure 2).

## DISCUSSION

This retrospective analysis was performed in order to compare outcomes from EGFR TKI as first- or second-line therapy for advanced NSCLC patients with sensitive EGFR mutations. The results demonstrated that first-line TKI therapy provided superior RR and PFS than second-line TKI therapy, and patients receiving first-line TKI followed by second-line chemotherapy experienced a longer OS than those who received first-line chemotherapy followed by second-line TKI therapy.

Several randomized clinical trials have been used to evaluate first-line TKI therapy for EGFR mutated NSCLC [7–12]. Current guidelines suggest first-line TKI therapy for this population, based on the results of superior PFS and RR of first-line TKI, compared to chemotherapy [16]. However, most of these studies failed to demonstrate improvement in OS. This raises the question of whether TKI is more effective in EGFR mutated NSCLC as a first-line therapy or is equally effective when administered as a second-line therapy [15]. In the IPASS, WJTOG 3405, NEJ002, and OPTIMAL (CTONG-0802) studies, the ORR of first-TKI therapies were 64.3%, 62.1%, 73.7%, and 82.9%, respectively [7, 8, 10, 11]. Due to limited sample size, the results of ORR for second-line TKI therapy for EGFR mutated NSCLC in previous clinical trials differed greatly. Nineteen EGFR mutated patients in the INTEREST study achieved an ORR of 42.1% [17]. Twenty-six EGFR mutated patients from the ISEL study showed a tumor response rate of 37.5% [18]. A meta-analysis summarized TKIs as different line therapies for NSCLC patients with EGFR mutations, and the results demonstrated that the response rate was 70% in first-line trials, while in three second-line trials the response rate was 47.4% [19]. In the present study, first-line TKI therapy achieved an ORR of 67.8%, which was significantly better than that achieved by second-line TKI therapy (55.6%). Similar results were reported in the NEJ002 study, which demonstrated that the response rate of EGFR-TKI decreased from 73.3% with first-line treatment to 58.5% in second-line treatment [7]. The median PFS of first-line TKI therapy in the current study was consistent with previous reports [20]. Compared with second-line, first-line TKI therapy provided longer PFS. Similar results were achieved in a combined survival analysis, which demonstrated that first-line TKI therapy provided a better PFS than second- or third-line TKI therapy (HR = 0.57; *P* = 0.007) [21]. Previously, a preclinical study used EGFR mutant NSCLC cell lines to explore whether prior exposure to platinum agents would affect subsequent responses to TKI therapy. The results suggested that first-line chemotherapy might reduce the benefit of subsequent EGFR-TKI treatment [22].

The high crossover rate to second-line or third-line EGFR TKI therapy in the first-line chemotherapy cohort could explain the failure to achieve a statistically longer OS in the first-line EGFR TKI therapy cohort. The PFS benefit of first-line TKIs did not appear to translate into an OS benefit in previous clinical trials. This could be partly explained by the subsequent effect of EGFR TKI therapy on OS. In the WJTOG 3405 study, more than 90% of the patients from the chemotherapy arm received EGFR TKI as a salvage therapy [13]. In the EURTAC study, the HR of OS for the first-line erlotinib arm versus the first-line chemotherapy arm was 0.92 (95% CI, 0.63–1.35). However, after using statistical models to control for second-line post-study treatment effects, the HR for OS was 0.68 (95% CI, 0.37–1.25) [14]. Similarly, the OPTIMAL study final OS results demonstrated that the median OS between the first-line erlotinib arm and the first-line chemotherapy arm was similar [23]. According to the in-depth analysis of the OPTIMAL study, 36.6% of the patients with common mutations that received first-line erlotinib did not receive post-study therapy, and 22.2% of the patients who received chemotherapy did not receive any post-study treatment. This could partly explain why the first-line erlotinib arm did not show superiority in OS over the first-line chemotherapy arm. Unlike previous reports of the reversible EGFR TKIs, erlotinib and gefitinib, the combined analyses of LUX-Lung 3 and LUX-Lung 6 studies demonstrated that first-line irreversible TKI afatinib provided a longer OS for common EGFR mutated NSCLC patients compared with chemotherapy [24]. In those two studies, very few patients in the first-line chemotherapy cohorts received afatinib as a subsequent treatment, because afatinib was not clinically available at the time of the studies, so most of the patients received erlotinib or gefitinib after chemotherapy [25, 26]. According to the results of the Lux Lung 7 study, afatinib might provide a longer PFS compared with gefitinib in treatment-naive patients with EGFR-mutated NSCLC [27].

In the present study, the OS analyses were conducted between two defined groups of patients: patients treated in sequence with first-line chemotherapy, followed by TKI in the second-line (CT-TKI), and patients treated with first-line TKI, and chemotherapy in the second-line (TKI-CT). The OS for the TKI-CT group (30.7 months) was longer than that of the CT-TKI group (27.2 months) (HR, 0.69; *P* = 0.02). These results were consistent with a previous study, which demonstrated that median OS in the gefitinib group was 7 months longer than that of the chemotherapy group (all patients were given gefitinib as the second-line treatment) [7]. Previously, the TORCH study compared first-line EGFR TKI followed by chemotherapy with first-line chemotherapy followed by second-line EGFR TKI. Subgroup analyses showed that patients with EGFR mutations experienced a greater benefit from first-line EGFR TKI followed by second-line chemotherapy [28]. The results of health-related quality of life (QoL) studies in previous clinical trials also supported first-line TKI therapy. The QoL of patients receiving first-line EGFR TKI was better than that of patients receiving first-line chemotherapy [7, 11, 29]. In the results of the LUX lung 3 compared with cisplatin/pemetrexed, first-line afatinib prolonged the time of deterioration of cough and dyspnea symptoms [25]. Some experts suggested that if TKIs were administered as a second- or third-line treatment, patients might miss the best opportunity to receive treatment with TKIs, because of a rapidly progressive disease during or after the first-line treatment [15].

A major limitation of this study is its retrospective nature. We could not collect the data of patients' QoL in the present study. In addition, due to the limited database, the ORR and PFS of second-line chemotherapy could not be determined. We could not compare the efficacy of first-line and second-line chemotherapy in the present study. According to the NEJ002 study, prior EGFR TKI therapy would not influence the efficacy of subsequent chemotherapy in patients with EGFR mutant NSCLC [7]. However, another study demonstrated a reduced sensitivity of subsequent chemotherapy compared with that of TKI-naïve frontline chemotherapy [30]. These results warrant more investigation.

In conclusion, compared with second-line TKI, first-line therapy achieved a longer PFS and a higher ORR in advanced NSCLC patients harboring sensitive EGFR mutations. The therapeutic strategy of using TKIs followed by chemotherapy can achieve longer OS than that using chemotherapy followed by TKIs, therefore this strategy should be considered as an optimal treatment option.

## MATERIALS AND METHODS

### Study design and patients

This study was designed to compare first- and second-line EGFR TKI therapies in patients with sensitive EGFR mutations. The study was approved by the Institutional Review Board of the Shanghai Chest Hospital. All of the patients were diagnosed with advanced NSCLC (stage IV) at the Shanghai Chest Hospital between January 2009 and December 2013. Baseline clinical characteristics included age at diagnosis, tumor histology, smoking history, and sex. We also abstracted treatment details, such as first- and second-line therapies, and the dates at which each line of therapy started. The inclusion criteria were (1) a pathologically confirmed advanced NSCLC, (2) a sensitive EGFR mutation consisting of an exon 19 deletion or an exon 21 point mutation (L858R), and (3) EGFR TKI therapy. Patients without survival and therapy details were excluded. The primary outcome was OS.

### Test method for EGFR mutations

DNA was extracted from five serial slices of a 5-μm paraffin section using the DNA FFPE Tissue Kit (Qiagen, Hilden, Germany). The highly sensitive method termed Amplification Refractory Mutation System (ARMS) was used to detect mutations in the EGFR gene according to the manufacturer's protocol of the DxS EGFR mutation test kit (DxS) [31].

### Statistical methods

For descriptive purposes, demographic and clinical data are summarized as medians with ranges for continuous variables, and categorical variables are expressed as summarized by the means of absolute and percentage numbers. The associations between patient demographics were examined using Pearson's χ^2^ test. Survival results are summarized as median values and two-sided 95% confidence intervals (CIs), and were analyzed using Kaplan–Meier analyses, whereas the log-rank test was used for comparisons among subgroups. Multivariable adjusted hazard ratios (HRs) for all-cause mortality by patient and treatment pattern were estimated using Cox regression. HRs were calculated along with their corresponding 95% CIs as measurements of association. Statistical significance was defined as *P* < 0.05. SPSS software, version 22 (SPSS Inc., Chicago, IL, USA) was used for all statistical analyses.

## SUPPLEMENTARY MATERIALS TABLES


