# Supplementary material for: Comparison of outcomes of tyrosine kinase inhibitor in first- or second-line therapy for advanced non-small-cell lung cancer patients with sensitive EGFR mutations
Source: Oncotarget. 2016 Sep 15;7(42):68442–8. doi: 10.18632/oncotarget.12035 (PMC5356566; doi:10.18632/oncotarget.12035)
Supplement: Supplementary file 1 [file oncotarget-07-68442-s001.pdf]

Comparison of outcomes of tyrosine kinase inhibitor in first- or second-line therapy for advanced non-small-cell lung cancer patients with sensitive EGFR mutations

Supplementary Materials

Supplementary Table S1: Demographic data of patients in CT-TKI and TKI-CT cohorts

Characteristic	TKI-CT (*n* = 141)	CT-TKI (*n* = 187)	*P*
Median age (range)	61 (32–86)	61 (30–81)	
≥ 60	82 (58.2%)	103 (55.1%)	0.578
< 60	59 (41.8%)	84 (44.9%)	
Gender			
Male	52 (36.9%)	84 (44.9%)	0.143
Female	89 (63.1%)	103 (55.1%)	
Smoking status			
Smoker	29 (20.6%)	45 (24.1%)	0.453
Never-smoker	112 (79.4%)	142 (75.9%)	
Histology			
Adeno	137 (97.2%)	161 (86.1 %)	0.001
Others	4 (2.8%)	26 (13.9%)	
Types of EGFR TKI			
Erlotinib	41 (29.1%)	38 (20.3%)	0.045
Gefitinib	74 (52.5%)	95 (50.8%)	
Icotinib	26 (18.4%)	54 (28.9%)	
Mutation type			
19 del	72 (51.1%)	94 (50.3%)	0.886
21 L858R	69 (48.9%)	93 (49.7%)	
PS			
0–1	134 (95.0%)	183 (97.9%)	0.159
≥ 2	7 (5.0%)	4 (2.1%)	
Chemotherapy regimens			
Platinum-based	102 (72.3%)	169 (90.4%)	< 0.001
Single-agent	34 (24.1%)	16 (8.6%)	
Others	5 (3.5%)	2 (1.1%)	
Third-line therapy			
Yes	67 (47.5%)	80 (42.8%)	0.393
No	74 (52.5%)	107 (57.2%)	

Supplementary Table S2: The regimens of chemotherapy in CT-TKI and TKI-CT cohorts

Chemotherapy agent	CT-TKI (*n* = 187)	TKI-CT (*n* = 141)
Platinum-based		
Platinum + Pemetrexed	85 (45.5%)	61 (43.2%)
Platinum + Gemcitabine	48 (25.7%)	22 (15.6%)
Platinum + Paclitaxel	11 (5.9%)	3 (2.1%)
Platinum + Docetaxel	17 (9.1%)	15 (10.6%)
Platinum + Vinorelbine	7 (3.7%)	0 (0.0%)
Platinum + Vinorelbine + IFO	1 (0.5%)	0 (0.0%)
Platinum + Pemetrexed + CCNU	0 (0.0%)	1 (0.7%)
Single agent		
Pemetrexed	9 (4.8%)	23 (16.3%)
Gemcitabine	3 (1.6%)	4 (2.8%)
Docetaxel	4 (2.1%)	7 (5.0%)
Others		
Pemetrexed + CCNU	2 (1.1%)	5 (3.5%)
